# 

**Published:** 1903-12-15

**Authors:** 


### CONTENTS.

What Shall We Do About Porcelain,
*By Edgar A. Honey, D.D.S.* 595
Practical Points in Practice, - *By C. C. Noble, D.D.S.* 604
The Lower Denture, - - *By R. C. Brophy, D.D.S.* 610
Cavity Linings, - - *By B. L. Thorpe, D.D.S.* 620
Ethics, - - - - - *By Dr. E. A. Bogue* 623
Conservative Treatment of the Antrum of Highmore,
*By Preston M. Hickey, M.D.* 627
EDITORIAL, - - - - - - - - - - 628
BIBLIOGRAPHY, - - - - - - - - 629
ANNOUNCEMENTS, - - - - - - - - - 631
INDENTS, - - - - - - - - - - 634

